# Implications of COVID-19 and Lockdown on Internet Addiction Among Adolescents: Data From a Developing Country

**DOI:** 10.3389/fpsyt.2021.665675

**Published:** 2021-05-12

**Authors:** Kristiana Siste, Enjeline Hanafi, Lee Thung Sen, Belinda Julivia Murtani, Hans Christian, Albert Prabowo Limawan, Levina Putri Siswidiani, Adrian

**Affiliations:** Department of Psychiatry, Faculty of Medicine, Universitas Indonesia – dr. Cipto Mangunkusumo General Hospital, Jakarta, Indonesia

**Keywords:** coronavirus disease 2019, internet addiction, lockdown, physical distancing, psychopathology, sleep quality, adolescents, Indonesia

## Abstract

**Introduction:** Physical distancing policy during coronavirus disease 2019 (COVID-19) pandemic requires adolescents to spend most of their time at home, thus increasing Internet use duration. Limited social interaction with their peers may lead to loneliness and an increased risk of mental health among adolescents. This study aimed to assess the prevalence of Internet addiction (IA) among adolescents and analyze the influence of psychosocial factors toward the heightened risk of IA during the COVID-19 pandemic.

**Methods:** An online survey comprising sociodemographic questionnaire, Internet Addiction Diagnostic Questionnaire (KDAI), Strengths and Difficulties Questionnaire (SDQ), and Pittsburgh Sleep Quality Index (PSQI) was distributed. Overall, a total of 2,932 adolescents (mean age, 17.38 ± 2.24 years old; female, 78.7%), originating from 33 of 34 provinces in Indonesia, completed the survey.

**Results:** The point prevalence of IA among Indonesian adolescents during the COVID-19 outbreak was 19.3%. Increased Internet use duration, internalization, externalization, low prosocial behavior, and sleep disturbances were found as risk factors of IA, either directly or as mediating variables. Physical distancing, large-scale social restriction (PSBB), and health status were not correlated to IA.

**Discussion:** Physical distancing was not established as a risk of IA. This could be due to other psychological factors such as internalization, externalization, prosocial, and sleep problems that had correlations to IA occurrence among adolescents in the COVID-19 pandemic. Sleep impairment might have resulted from the emotional and behavioral issues and directly contributed to IA development.

**Conclusion:** The present study found the prevalence of IA among Indonesian adolescents to be higher than the adult during the COVID-19 pandemic. Several psychological measures were indicated to increase the risk of IA, while physical distancing did not elevate the risk. Thus, remote schooling is preferable in Indonesia along with proper parental supervision to minimize Internet use for entertainment purposes.

## Introduction

The coronavirus disease 2019 (COVID-19) outbreak has brought about detrimental impacts on all members of society. Following the exponential growth of COVID-19 cases, physical distancing became mandatory in many countries, including Indonesia, as WHO suggested. Physical distancing in Indonesia is known as PSBB (*Pembatasan Sosial Berskala Besar*—large-scale social restriction) (1–4). This policy creates some significant social and behavioral changes, especially among adolescents (5–7). The implementation of school from home or distance learning, locally known as PJJ (*Pembelajaran Jarak Jauh*) has started in March 2020 until now. The virtual schooling could last for an unspecified amount of time, as currently, the COVID-19 cases kept increasing in Indonesia. This delicate circumstance also has led to the restriction of recreational activities (4). Physical distancing enforces adolescents to spend most of their time at home, thus increasing the duration of Internet use, not only for academic reasons but also for playing video games and social media (5–7). COVID-19 and the limited social interaction with their peers may contribute to loneliness and escalated risk of mental health problems such as depression, anxiety, traumatic experiences, and sleep disorder among adolescents (8–11). Adolescence is an intricate transition period before adulthood. The individual tends to spend more time and feels more comfortable expressing their feelings to their peers than their parents (12, 13). The relationship between adolescents and their peers is crucial, as they considered their peers as a source of affection, trust, inclusivity, and self-esteem (14). In addition, the demand to rapidly adapt to the new COVID-19 norms puts an additional psychological burden on adolescents. Some experts argued that implementing the abrupt virtual learning method can decrease study motivation, neglect daily activities, and a rise in dropouts (15). Surveys from Hong Kong and China demonstrated an increase in stress and anxiety among students during the COVID-19 pandemic due to uncertainty in various academic activities such as exams, exchange programs, and graduation (10). Financial burden was another source of stress, as some students lost their part-time jobs, and at the same time, their families struggled with unstable income and lay-offs during the pandemic (10, 15, 16).

During the COVID-19 pandemic, there had been an increase in Internet use in Indonesia, exemplified by the rise in national mobile data traffic by up to 16.1% (17, 18). With the ban on physical contacts, almost all daily activities had transitioned into the digital realm (7, 19, 20). The Internet also plays a vital role in disseminating information about COVID-19, primarily through social media (7, 19, 21). A prior study in China showed that 82% of subjects used social media more frequently during the COVID-19 pandemic to keep themselves updated (19); however, the lack of regulation regarding information distribution by the government would create a loophole for rampant hoaxes and further anxiety within the society (9, 22). Immature cognitive functioning, compared to impulse processing, in adolescents diminishes their ability to appraise information appropriately. Thus, they are prone to false information and negative emotion during times such as this pandemic (13, 22). Anxiety and depression may induce preoccupation with using social media and games to modify these negative moods (19–21). The maladaptive coping mechanism could progress into excessive Internet use and increase the risk of Internet addiction (IA) if left untended (7, 20).

The present study aimed to investigate the rate of IA among adolescents and the influence of psychosocial factors toward the heightened risk of IA during the COVID-19 pandemic. The results can be considered in spurring and guiding a national policy pertaining to mental health and addiction, especially on IA during and after the COVID-19 outbreak.

## Methods

### Sample and Procedure

The authors produced an online survey using *Google Form*. After clicking the survey link, the survey started with a title page containing an outline of the study's purpose, respondents' criteria, and data management. Each respondent was asked for informed consent, and an author's email for correspondence was provided for further inquiries. Those who did not provide consents were directed to finish without filling out the survey. The survey contained a sociodemographic section (gender, age, monthly household income, occupations, province of residence, and education level), then followed by quarantine-related questions (the practice of quarantine and physical distancing, location of quarantine, living companion during quarantine, and confirmed/suspected cases within the household), and Internet usage characteristics (perceived Internet duration change, duration during and before quarantine, motives, the age when the Internet was first used, and frequent social media applications or game genres). In the last section, each respondent was asked to complete the Internet Addiction Diagnostic Questionnaire (KDAI), Strengths and Difficulties Questionnaire (SDQ), and Pittsburgh Sleep Quality Index (PSQI). The survey was separated into 14 sections (with several instruments were divided into more than one section) and required about 40–50 min for completion, although response time could not be evaluated in *Google Form* to prevent reporting bias. All questions were marked, and mandatory before respondents could proceed to the next section or submit the survey.

Physical distancing as an extension of self-quarantine included several practices defined in this study as studying/working from home, alternate studying/working days, and/or other physical distancing practices as per the guideline from Indonesian COVID-19 Response Acceleration Task Force (GTPP COVID-19). Respondents were asked whether they and/or any household member had been declared as COVID-19 suspect cases and/or diagnosed with COVID-19, following the descriptions provided by the GTPP COVID-19, Indonesian Ministry of Health, and World Health Organization. Province of residence was categorized into whether PSBB had been implemented at the commencement of the study (April 28, 2020) based on data from GTPP Covid-19, which included DKI Jakarta, West Java, East Java, Central Java, Banten, West Kalimantan, North Kalimantan, Gorontalo, West Sumatera, Riau, and South Sulawesi. Income levels were divided based on classification by the World Bank.

A shortened hyperlink was devised and publicized by the research team to schools, teachers, and parental groups as contact points between April 28 (44 days since stay-at-home notification and 18 days since PSBB) and June 30, 2020. All contact points were suggested to pass on the survey link to others. Parents, guardians, and teachers were asked to pass on the survey link to the adolescents if they agreed to allow them to participate. Enrolled respondents (i) were asked to provide emails (names were not requested) to prevent multiple responses; they should be (ii) aged 10–20 years old, (iii) currently residing in Indonesia, and (iv) capable of understanding Bahasa Indonesia. Age range was selected based on the combination of WHO definition of 10–19 years old (23) and the Indonesian Pediatric Association of 10–20 years old (24) for adolescence. Responses that were non-consenting (*n* = 30), duplicates (*n* = 23), and from those currently not residing in Indonesia (*n* = 10) were removed. About 40 respondents answered the Symptoms Checklist 90 (SCL-90), which was provided for the adult (≥21 years old) demographic (this survey was part of a more extensive study simultaneously targeting adults), instead of SDQ, and were removed from all analyses. Personal information (e.g., emails) was only accessible to researchers. They were only inspected for duplicates and dropped before further data examination; thus, the research team could not link the data and respondent. Overall, a total of 2,932 respondents completed the survey encompassing 33 of 34 provinces in Indonesia and seven main islands (Java, 78.5%; Sumatera, 8.3%; Kalimantan, 0.6%; Sulawesi, 9.7%; Nusa Tenggara and Bali, 2.6%; Papua, 0.1%; and Maluku, 0.2%) across Indonesia.

### Instruments

#### Internet Addiction Diagnostic Questionnaire

KDAI was developed in Indonesia with excellent reliability (α = 0.942), sensitivity (91.8%), and negative likelihood ratio (0.11). The instrument is self-administered with a total of 44 statements composed of 7 subscales, i.e., withdrawal (8 items), loss of control (9 items), priority enhancement (6 items), negative consequences (7 items), mood modification (5 items), salience (6 items), and impairment (3 items). Each statement has a 7-point Likert scale, 0 (=not applicable), 1 (= very rarely), 2 (= rarely), 3 (= sometimes), 4 (= often), 5 (= very often), and 6 (= always). A score of ≥108 indicates internet addiction (with a maximum score of 264). Each domain's reliability was satisfactory, Cronbach's alphas were between 0.641 and 0.933, and overall α = 0.979.

#### Strengths and Difficulties Questionnaire

SDQ is a questionnaire for children age 4–17 years old (25) and has been validated within the youth population (26). The questionnaire consists of 25 items regarding children's behavior in the past 6 months. Those items are divided into five subscales: hyperactivity, emotional symptoms, conduct problems, peer problems, and prosocial. Each item is marked with “Not True” (= 0), “Somewhat True” (= 1), and “Certainly True” (= 2). Scores of “Not True” and “Somewhat True” are reversed for the prosocial behavior subscale. The total score for each subscale is generated by summing the scores for the five items, thereby resulting in a composite score ranging from 0 to 10 (27). Within the general population, it has been hypothesized that a three-factorial SDQ performs better than the five-factor structure. This study utilized the three-factor SDQ concerning the generalized population of the target respondents. The sum of the conduct and hyperactivity scales resulted in externalization scores, ranging from 0 to 20. In contrast, the internalization subscale was generated by the sum of the emotional and peer problems scales, also ranges from 0 to 20 (28). Score for SDQ scores are divided into four bands, namely, 80% “close to average,” 10% “slightly raised/lowered,” 5% “high/low,” and 5% “very high/very low;” externalization and internalization were classified according to the samples' distribution, and prosocial subscales employed the cutoff points provided by the tridimensional validation study (28) and another study among younger and older Indonesian adolescents (29). The Indonesian version of SDQ has a sensitivity of 67% and a specificity of 68%, with α = 0.773 (27).

#### Pittsburgh Sleep Quality Index

The PSQI is a commonly used instrument to assess sleep quality on clinical or nonclinical subjects with the reliability in a previous study of α = 0.845 (30, 31). The questionnaire consists of 24 items, divided into 20 multiple choices and 4 open-ended questions. About 5 of 24 items need assessment from a partner or another individual on the subject's sleep pattern. Another 19 items were self-answered questions and can be grouped into seven components, with each being measured between 0 and 3 (maximum 21). A score >5 indicates poor sleep quality. The Indonesian version of PSQI has been validated with reliability of α = 0.79, content validity of 0.89, and specificity of 81% (32).

### Statistical Analysis

The study employed SPSS version 23.0 for Windows for the majority of statistical tests. Age, duration of Internet use, and scores of all instruments were compared using the Mann–Whitney U test due to the non-normal distribution. A hierarchical multiple regression analysis was performed using three blocks; block one contained only sociodemographic and quarantine variables, block two was appended with Internet usage characteristics, and block three was added with results of the three psychometric tools. *P* < 0.05 was a consideration for significance along with the appropriate (not crossing 1 for ratios or 0 for difference) 95% confidence intervals (CIs). AMOS 24.0 was utilized to compute the path analysis, which was chosen to complement regression analysis since previous data highlighted the possibility of disparity (33). Path analysis employed a directed mediation relationship. Still, it was not utilized to assess causality, as it can only be established through a suitable study design. No single statistical model can prove causality without the former criteria being met (34). In determining the total, direct, and indirect effects of all independent and mediator variables, bootstrapping (5,000 iterations) and 95% bias-corrected CI were applied. Good fit was defined as root mean square error of approximation (RMSEA) <0.05, standardized root mean square residuals (SRMR) <0.06, comparative fit index (CFI) >0.95, and goodness-of-fit index (GFI) >0.95, while RMSEA <0.08, SRMR <0.09, CFI >0.90, and GFI >0.90 constituted acceptable fit (35).

### Ethical Approval

The study received ethical clearance from the institutional review board of Faculty of Medicine, Universitas Indonesia—dr. Cipto Mangunkusumo General Hospital (Ref: KET-413/UN2.F1/ETIK/PPM/00/02/2020).

## Results

### Descriptive Analysis

On average, the respondents were 17.38 years old, and about 78.7% were female. Of 2,932, 565 (19.3%, 95% CI 17.9–20.7%) respondents met the study's criteria of IA, and among those, about 75.2% (*n* = 425) were female. Comparing between respondents with IA and without, there were significant differences in the distribution of age of first Internet use, usage motives, PSQI, and SDQ (shown in Tables 1, 2). Around half of the non-IA respondents had started using the Internet by the age of 12 and 11 for IA samples. Duration of Internet use increased for both groups of respondents alike. On average, there was a 59.7% increase in the duration of Internet usage from before (7.27 h) to during (11.61 h) the COVID-19 pandemic.

**Table 1:** Descriptive psychometric and demographic data of respondents by KDAI classification.

**Variables**	**KDAI**				
	**IA (*N* = 565)**	**Normal (*N* = 2,307)**	**U^a^**	**Total (*****N*** **= 2,932)**	
	**Median (IQR)**	**Median (IQR)**		**Mean ± SD**	**Median (IQR)**
Age	17 (16,19)	18 (16,19)	1.53	17.38 ± 2.24	18 (16,19)
First Age of Internet Use	11 (9,3)	12 (10,14)	7.41^***^	11.71 ± 2.55	12 (10,13)
Internet Duration During COVID-19	12 (8,16)	11 (6.75, 15)	−4.15^***^	11.61 ± 6.14	12 (7,15)
Internet Duration Before COVID-19	6 (4.5, 10)	6 (4,10)	−2.05^*^	7.27 ± 4.89	6 (4,10)
PSQI	6 (4,9)	5 (3,7)	−10.47^***^	5.65 ± 2.98	5 (3,7)
Internalization	9 (6,11)	6 (4,9)	−13.56^***^	6.94 ± 3.79	7 (4,10)
Externalization	7 (5,9)	5 (3,7)	−14.49^***^	5.51 ± 2.93	5 (3,7)
Prosocial	8 (6,9)	8 (7,10)	6.23^***^	7.70 ± 2.41	8 (7,10)
KDAI	130 (118, 143)	64 (47, 84)	−36.99^***^	76.29 ± 39.30	73 (51, 98)

a *Mann–Whitney U-test statistic*.

* *p < 0.05*;

*** *p < 0.001*.

*IQR, interquartile range; SD, standard deviation*.

**Table 2:** Sociodemographic and COVID-19-related variables stratified by Internet addiction.

**Variables**	**KDAI**			
	**IA (*N* = 565)**	**Normal (*N* = 2,307)**	**χ2^a^**	**Total (*N* = 2,932)**
	***N* (%)**	***N* (%)**		***N* (%)**
**Sex**				
Male	140 (24.8)	485 (20.5)	5.00^*^	625 (21.3)
Female	425 (75.2)	1,882 (79.5)		2,307 (78.7)
**Positive/suspect cases within household**				
No	542 (95.9)	2,287 (96.6)	0.64	2,829 (96.5)
Yes	23 (4.1)	80 (3.4)		103 (3.5)
**Perceived internet use change**				
Increased	527 (93.3)	2,101 (88.8)	10.97^**^	2,628 (89.6)
Decreased	13 (2.3)	67 (2.8)		80 (2.7)
Unchanged	25 (4.4)	199 (8.4)		224 (7.6)
**Main Internet usage motives**				
Social media	166 (29.4)	551 (23.3)	26.73^***^	717 (24.5)
Games online	26 (4.6)	60 (2.5)		86 (2.9)
Information seeking	61 (10.8)	246 (10.4)		307 (10.5)
Shopping	1 (0.2)	3 (0.1)		4 (0.1)
Entertainment	28 (5.0)	94 (3.2)		122 (4.2)
Virtual relationship	0 (0)	1 (0)		1 (0)
Online gambling	1 (0.2)	0 (0)		1 (0)
Online pornography	0 (0)	0 (0)		0(0)
Work/Education/Online training	282 (49.9)	1412 (59.7)		1,694 (57.8)

a *Chi-square test*.

* *p < 0.05*;

** *p < 0.01*;

*** *p < 0.001*.

### Influences of Sociodemographic, Internet Usage Attributes, and Psychopathologies Toward Internet Addiction

The hierarchical models displayed a significant Δ*F* test on consequent blocks with a final R^2^ of 0.191. As displayed in Table 3, biological age did not correlate to KDAI scores. Gender was significantly correlated with attainment of senior high school education, and nearly all respondents (96.3%) are still students, with the majority being university students. A larger proportion of respondents were within the lower half of the income brackets, with 35.8% reporting low monthly household income and 44.2% within the lower-middle band. Most (84.9%) of the subjects lived in areas implementing PSBB, and 96.1% practiced physical distancing; furthermore, 103 reported having suspected/confirmed cases within their households. More than half (57.8%) of the respondents reported mainly using the Internet for work or academic purposes. Compared to this, 24.5% of those who surfed the Internet for social media was significantly associated to IA (B: 4.32; 95% CI 1.04–7.59, *p* < 0.01). Heightened risk toward IA (B: 12.12; 95% CI 3.73–20.52, *p* < 0.01) was also observed among 86 (2.9%) respondents who primarily utilized the Internet for gaming.

**Table 3:** Regression analysis between sociodemographic, Internet usage characteristics, PSQI, and SDQ with KDAI during COVID-19 pandemic.

**Variables**	***N* (%)**	**Model 1**	**Model 2**	**Model 3**
		**B (95% CI)**	**B (95% CI)**	**B (95% CI)**
Age	–	−0.62 (−1.60, 0.36)	0.08 (−0.91, 1.07)	−0.02 (−0.96, 0.93)
**Gender**				
Female	2,307 (78.7)	Reference		
Male	625 (21.3)	6.34^***^ (2.77, 9.90)	2.56 (−1.57, 6.69)	3.33 (−0.62, 7.29)
**Education**				
Up to Junior High school	895 (30.5)	Reference		
Senior High school	1,829 (62.4)	−1.92 (−6.53, 2.69)	−0.90 (−5.38, 3.58)	−1.33 (−5.61, 2.94)
Higher Education	208 (7.1)	−2.79 (−9.81, 4.23)	0.88 (−5.93, 7.69)	1.87 (−4.63, 8.37)
**Occupation**				
Students	2,824 (96.3)	Reference		
Professionals	74 (2.5)	−3.29 (−12.46, 5.88)	−1.05 (−10.02, 7.93)	0.20 (−8.36, 8.75)
Homemakers	11 (0.4)	13.04 (−10.03, 36.12)	11.25 (−11.13, 33.62)	9.63 (−11.66, 30.92)
Office Workers/Proprietors	11 (0.4)	1.58 (−21.59, 24.75)	2.03 (−20.44, 24.50)	5.59 (−15.81, 26.99)
Unemployed	12 (0.4)	34.45^**^ (12.36, 56.53)	28.72^**^ (7.36, 50.08)	25.48^*^ (5.12, 45.85)
**Household Income**				
Low	1,049 (35.8)	Reference		
Lower Middle	1,297 (44.2)	3.66^*^ (0.48, 6.84)	0.58 (−2.55, 3.72)	0.87 (−2.12, 3.87)
Upper Middle	434 (14.8)	11.33^***^ (6.96, 15.70)	1.50 (−3.07, 6.08)	1.93 (−2.42, 6.29)
High	152 (5.2)	12.23^***^ (5.58, 18.88)	−1.20 (−8.03, 5.63)	0.68 (−5.84, 7.21)
**Province**				
Do Not Implement PSBB	442 (15.1)	Reference		
Implement PSBB	2,490 (84.9)	0.079 (−4.04, 4.19)	−2.02 (−6.05, 2.01)	−0.62 (−4.46, 3.23)
**Physical distancing**				
Do Not Practice	113 (3.9)	Reference		
Practice	2,819 (96.1)	−0.59 (−7.94, 6.76)	−1.91 (−9.04, 5.21)	−0.31 (−7.12, 6.50)
**Suspected/Confirmed Cases of COVID-19 within Household**				
No	2,829 (96.5)	Reference		
Yes	103 (3.5)	4.62 (−3.04, 12.28)	2.93 (−4.49, 10.36)	1.84 (−5.23, 8.92)
**Perceived internet use change**				
No Change	224 (7.6)	Reference		
Increase	2,628 (89.6)	–	9.37^***^ (3.99, 14.76)	8.91^***^ (3.77, 14.04)
Decrease	80 (2.7)	–	10.08^*^ (0.35, 19.81)	6.31 (−2.97, 15.59)
**Internet usage during COVID-19**	–	–	0.59^***^ (0.25, 0.93)	0.38^*^ (0.05, 0.70)
**Internet usage before COVID-19**	–	–	−0.16 (−0.58, 0.26)	−0.02 (−0.42, 0.38)
**Age of first internet use**	–	–	−1.94^***^ (−2.59, −1.30)	−1.77^***^ (−2.39, −1.16)
**Internet monthly spending^a^**				
<50,000	224 (7.6)	Reference		
50,000–100,000	978 (33.4)	–	0.98 (−4.61,6.58)	2.30 (−3.03, 7.63)
100,000–150,000	565 (19.3)	–	2.65 (−3.44, 8.74)	4.75 (−1.06, 10.55)
150,000–200,000	0 (0)	–	0	0
200,000–250,000	213 (7.3)	–	6.03 (−1.27, 13.34)	5.70 (−1.26, 12.66)
>250,000	952 (32.5)	–	2.96 (−2.79, 8.70)	4.42 (−1.06, 10.55)
**Main internet usage motives**				
Social Media	717 (24.5)	–	5.40^**^ (1.97,8.83)	4.32^**^ (1.04, 7.59)
Games Online	86 (2.9)	–	14.56^***^ (5.76, 23.37)	12.12^**^ (3.73, 20.52)
Information Seeking	307 (10.5)	–	4.02 (−0.62, 8.67)	3.33 (−1.09, 7.76)
Shopping	4 (0.1)	–	11.35 (−25.67, 48.36)	9.75 (−25.53, 45.02)
Entertainment	122 (4.2)	–	2.34 (−4.80, 9.48)	2.41 (−4.40, 9.21)
Virtual Relationship	1 (0)	–	−76.61^*^ (−151.09, −2.14)	−92.30^*^ (−163.36, −21.24)
Online Gambling	1 (0)	–	84.04^*^ (9.50, 158.59)	67.09 (−4.10, 138.27)
Online Pornography	0 (0)	–	0	0
Work/Education/Online Training	1,694 (57.8)	Reference		
**PSQI**				
Normal	1,604 (54.7)	Reference		
Sleep Problems	1,328 (45.3)	–	–	8.15^***^ (5.38, 10.91)
**SDQ**				
**Internalization**				
Close to Average	2,402 (81.9)	Reference		
Slightly High	274 (9.3)	–	–	12.68^***^ (8.01, 17.34)
High	149 (5.1)	–	–	18.21^***^ (11.99, 24.42)
Very High	107 (3.6)	–	–	17.24^***^(9.16, 25.31)
**Externalization**				
Close to Average	2,264 (77.2)	Reference		
Slightly High	376 (12.8)	–	–	13.90^***^ (9.79, 18.02)
High	190 (6.5)	–	–	12.26^***^ (6.70, 17.81)
Very High	102 (3.5)	–	–	15.26^***^ (7.08, 23.45)
**Prosocial**				
Close to Average	2204 (75.2)	Reference		
Slightly Low	280 (9.5)	–	–	8.93^***^ (4.44, 13.43)
Low	176 (6.0)	–	–	7.91^**^ (2.30, 13.52)
Very Low	272 (9.3)	–	–	7.38^**^ (2.75, 12.01)
**ΔR**^**2**^		0.022	0.081	0.088
***ΔF***		5.58^***^	7.95^***^	31.30^***^

a *In Indonesian rupiahs*.

* *p < 0.05*;

** *p ≤ 0.01*;

*** *p ≤ 0.001*.

Nearly 89.6% of the respondents subjectively perceived that their Internet use surged during the pandemic, which was significantly correlated to a higher KDAI score (B: 8.9; 95% CI 3.77–14.04, *p* < 0.001), and so was the estimated duration of Internet use during COVID-19 (B: 0.38; 95% CI 0.05–0.70, *p* < 0.05). Report of sleeping disturbance (B: 8.15; 95% CI 5.38–10.91), *p* < 0.001) and all bands of externalization, internalization, and prosocial were linked to an increase in KDAI scores.

### Mediation Effects of Sociodemographic, SDQ, and PSQI Toward KDAI

**Figure 1:**
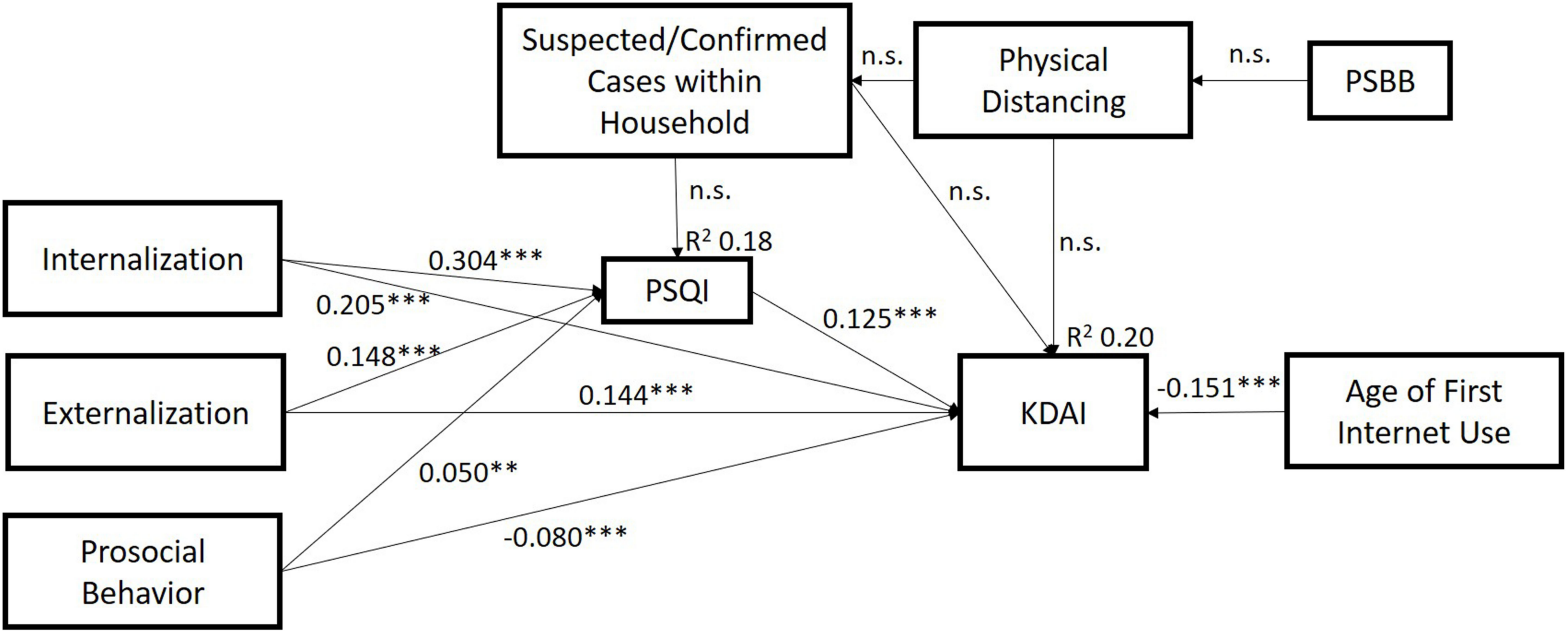
Model of significant path analysis among variables. Values represent the path coefficients. For clarity, some nonsignificant path coefficients (e.g., between suspected/confirmed COVID-19 cases and SDQ subscales) and control variables (age, gender, duration of Internet usage, and household income) are not shown in the figure. Gender [β = 0.072, *p* ≤ 0.001, 95% CI (0.038, 0.105)], household income [β = 0.044, *p* < 0.05, 95% CI (0.009, 0.084)], and duration of Internet usage [β = 0.070, *p* ≤ 0.01, 95% CI (0.022, 0.118)] were significantly correlated to Internet addiction while biological age was not.

To thoroughly test the relationships between variables, a path analysis (see Figure 1) was performed according to a predefined modeling and study aims. The model was of good fit with the data {χ^2^/df = 1.998; GFI, 0.998; CFI, 0.996; RMSEA = 0.018 [90% CI (0.011, 0.026)], SRMR = 0.010}. Biological age, gender, household income, and duration of Internet usage during and before COVID-19 were all controlled for in the model: gender [β = 0.072, *p* ≤ 0.001, 95% CI (0.038, 0.105)], duration of Internet usage during COVID-19 [β = 0.070, *p* ≤ 0.01, 95% CI (0.022, 0.118)], and household income band upper-middle [β = 0.044, *p* < 0.05, 95% CI (0.009, 0.084)] were significantly correlated to KDAI. All subscales of SDQ were correlated to KDAI directly [internalization: β = 0.205, *p* ≤ 0.001, 95% CI (0.161, 0.248); externalization: β = 0.143, *p* ≤ 0.001, 95% CI (0.102, 0.187); prosocial: β = −0.080, *p* ≤ 0.001, 95% CI (−0.120, −0.142)] and indirectly through PSQI [internalization: β = 0.039, *p* < 0.001, 95% CI (0.026, 0.053); externalization: β = 0.019, *p* ≤ 0.001, 95% CI (0.012, 0.029); prosocial: β = 0.081, *p* ≤ 0.01, 95% CI (0.002, 0.012)]. Age of first Internet use [β = −0.151, *p* ≤ 0.001, 95% CI (−0.189,−0.113)] were negatively linked to KDAI. The mediation model explained 20.3% of the variances of IA (KDAI) and 18.4% of sleep disturbance (PSQI).

## Discussion

This study observed a point prevalence of 19.3% for suspected IA among Indonesian adolescents during the COVID-19 outbreak. Additionally, the current study demonstrated that quarantine policies (i.e., PSBB) and other health practices were not correlated to IA's risk during the pandemic. We presented a significant correlation between increased Internet use duration, psychological measures, and sleep disturbances as risk factors and mediating variables to IA.

The current study was the first study of national scope in Indonesia regarding adolescent's IA rate; thus, comparing a figure before the pandemic was challenging. Nonetheless, the presented rate was higher than the prevalence among adults (14.4%) over the same period (36). This result was approximately in line with a Taiwanese study specifying a rate of 24.4% of IA among junior high school students (37) although astronomical than another study that reported 2.68% of Chinese adolescents screened with addictive Internet use during the COVID-19 pandemic (38). Notably, this study's finding was higher than the global average of 6% before the pandemic but similar to the aggregated rate among Southeast Asian adolescents, 19.6% (39). Nonetheless, other data of IA across Asia, Japan (6.2%) (40), South Korea (10.7%) (41), Taiwan (15.8%) (42), and China (0.2%) (43) demonstrated much lower rates before the pandemic and highlighted the rising trend over the COVID-19 pandemic course.

The majority of adolescents in this study reported living in provinces implementing PSBB, and nearly all disclosed that they practiced physical distancing. PSBB and physical distancing practice were neither directly nor indirectly, through sleep disturbance or other psychopathologies, correlated to IA. These patterns were concordant with previous findings among Indonesian adults (36). However, we observed neither a significant correlation of COVID-19 proximity to IA nor any psychopathologies, both of which were significant within the Indonesian adult population. This could be assumed due to differences in stress perception (immediate vs. distant) (44) and emotional regulation (45) to differing psychological stressors during the COVID-19 pandemic, which could be scrutinized in the future. Notably, the average age of first Internet use among children in European countries was estimated around 8 years old (46); meanwhile, among American adolescents, the mean age of first Internet use was about 9 years old (47). Unfortunately, the information on the age of first Internet use in Asia was scarce, with one available evidence among Singaporean children noted an average of similarly 8 years old (48). The mean age of first Internet use in this study was 11.71 ± 2.55 years old. Furthermore, our study found that earlier age of first Internet use was associated with a heightened risk of IA, mirroring the observations by several other studies (49–51). As an individual is introduced to the Internet earlier, they would be able to more seamlessly digitalize their daily lives and adopt broader modern technological domains (52), a trait that is, to a certain extent, mandatory and beneficial during the COVID-19 pandemic but may also present with psychological drawbacks.

Our findings showed that internalization and externalization problems were correlated to higher KDAI scores. This finding corresponded with other studies about this issue on adolescents (53–55). Our previous study before the COVID-19 pandemic on adolescence also showed a similar result (56). However, most studies were cross-sectional, which could not explain causality between internalization/externalization problems and IA (53, 55). A longitudinal study showed that internalization or externalization problems might contribute as risk factors for IA (54). Yet, further replicable results are required to confirm and explore how internalization and externalization problems among adolescents might influence IA. Generally, adolescents are more susceptible to develop IA than adults, even before the COVID-19 outbreak. The prefrontal cortex's immaturity results in insufficient executive and inhibition of cognitive control in adolescents, predisposing them to develop IA (57, 58). Additionally, the Internet provides instantaneous gratification, thus illusively aiding adolescents to evade their loneliness and negative mood and turning the Internet into a form of escapism (20, 59). Psychosocial stress occurring during COVID-19 quarantine measures such as lifestyle changes, economic burden, the bombardment of fearful news, and fear of contracting COVID-19 could lead to sleeping problems. In addition, PSBB entails the abrupt implementation of remote learning (PJJ), which removes the peer and physical connections pivotal for adolescent growth and social capital (60) and introduces tremendous pressure while adapting to their challenging situation and maintaining their academic goals (10, 15, 16). The mental buffer, coping mechanism, and social support of each adolescent, in turn, determine the degree of their psychological burden (61) (scores of SDQ) that correlated positively to IA susceptibility in this study. Adolescents might then be reinforced to use the Internet to modify their mood due to the COVID-19 stressors. Alternatively, the Internet can also become a source of virtual support with family and friends when use appropriately (11, 59).

Prosocial behavior score was directly negatively associated with IA in our study but was indirectly positively correlated to higher KDAI scores through PSQI. The former was in line with previous studies (62–64). Additionally, prolonged duration of Internet use is hypothesized to increase dopamine release and lead to addictive behavior (65, 66). Concurrently, our finding showed that certain motives of Internet use (i.e., social media and online games) were at increased odds to the risk of IA. Several studies have shown that online games, particularly competitive and violent games, were related to a lower prosocial behavior level (67, 68). Contrastingly, exposure to positive prosocial content on social media and games promoted a higher prosocial behavior level (69, 70). Therefore, the content, complementary to duration, of Internet use should be supervised and regulated by the parents, especially during the COVID-19 quarantine period, during which adolescents have diminished opportunities to socialize physically. Impairment of sleep was found in nearly half of the adolescents in this study, which might stem from the observed emotional and behavioral problems. Similarly, a prior study in Italy noted that poor sleep was more prevalent and negatively affected psychological well-being during the COVID-19 lockdown (71). Studies showed that dependence on the Internet was associated with sleep deprivation, decreased sleep quality, and increased sleep latency (72, 73). IA could drive adolescents to lose sleep as they continuously surf the Internet; conversely, sleeping problems might be attributed to their extended Internet use (73). The result of the path analysis demonstrated that sleep problems could also directly attribute to maladaptive Internet use in the adolescent population during the COVID-19 outbreak.

The current study found the prevalence of IA among Indonesian adolescents to be higher than the adult counterpart during the COVID-19 pandemic. Increased Internet use duration, externalizing and internalizing problems, decreased prosocial behavior, and sleep disturbances were found to increase the risk of IA, either directly or indirectly, as mediating variables during the pandemic. Yet, physical distancing, PSBB, and health status were not correlated to IA in this study. This was the first large sample study that covered nearly all provinces of Indonesia and investigated IA among adolescents. The study's data could provide vital information to establish national policy, particularly regarding internet use regulation for adolescents. Taken together, remote schooling is preferable in countries such as Indonesia, which saw upward and spiking of COVID-19 cases, along with appropriate supervision by parents to minimize internet use for entertainment purposes. The study possessed several limitations. First, this study collected data through the Internet, which posed selective and response biases. The sampling design did not employ randomization and, due to the timeframe, could not be designed with more sophisticated measures, e.g., respondent-driven sampling, which resulted in unbalanced sex distribution. The disproportionate distribution might also be attributed to the nature of participation being voluntary and differences in gender-associated inclinations (74). Nonetheless, most of the provinces in Indonesia were represented with appropriate weightage; for example, the Java island had the highest proportion. Thus, the geographical spread and large respondents provided ample foundations to explore correlations and interactions representatively. The psychometric instruments were all self-reported; therefore, limitation such as social desirability should be considered. Lastly, the study could not address any causal relationships between the variables, which should be explored in future research and compared to the present study.

## Data Availability Statement

The raw data supporting the conclusions of this article will be made available by the authors, without undue reservation.

## Ethics Statement

The studies involving human participants were reviewed and approved by Institutional Ethics Committee of Faculty of Medicine, Universitas Indonesia—dr. Cipto Mangunkusumo General Hospital (Ref: KET-413/UN2.F1/ETIK/PPM/00/02/2020). Written informed consent to participate in this study was provided by the participants' legal guardian/next of kin.

## Author Contributions

KS and EH designed and supervised the study. KS, EH, LTS, BM, and HC contributed data or analysis tools. KS, EH, LTS, BM, HC, APL, A, and LPS collected the data. KS, EH, LTS, HC, and BM performed the data analysis. KS, EH, LTS, BM, HC, APL, A, and LPS wrote the manuscript. KS secured funding for the study. All authors contributed to the article and approved the submitted version.

## Conflict of Interest

The authors declare that the research was conducted in the absence of any commercial or financial relationships that could be construed as a potential conflict of interest.

## References

[1] World Health Organization. Coronavirus Disease (COVID-19) Situation Report - 162. Geneva: World Health Organization. (2020).

[2] CNN Indonesia. Update Corona 30 Maret: 1.414 Kasus, 122 Meninggal, 75 Sembuh. (2020). Available online at: https://www.cnnindonesia.com/nasional/20200330113537-20-488187/update-corona-30-maret-1414-kasus-122-meninggal-75-sembuh (accessed May 30, 2020).

[3] World Health Organization. Physical Distancing. World Health Organization - Western Pacific Region. (2020). Available online at: https://www.who.int/westernpacific/emergencies/covid-19/information/physical-distancing (accessed Jun 24, 2020).

[4] Gugus Tugas Percepatan Penanganan COVID-19. Regulasi. (2020). Available online at: https://covid19.go.id/p/regulasi? (accessed May 20, 2020).

[5] Sun Y Li Y Bao Y Meng S Sun Y Schumann G. Brief report: increased addictive internet and substance use behavior during the COVID-19 pandemic in China. Am J Addi. (2020) 29:268–70. 10.1111/ajad.13066 32500608 PMC7300868

[6] Chao M Chen X Liu T Yang H Hall BJ. Psychological distress and state boredom during the COVID-19 outbreak in China: the role of meaning in life and media use. Eur J Psychotraumatol. (2020) 11:1769379. 10.1080/20008198.2020.1769379 33029315 PMC7473057

[7] King DL Delfabbro PH Billieux J Potenza MN. Problematic online gaming and the COVID-19 pandemic. J Behav Addi. (2020) 9:184–6. 10.1556/2006.2020.00016 32352927 PMC8939428

[8] Fiorillo A Gorwood P. The consequences of the COVID-19 pandemic on mental health and implications for clinical practice. Eur Psychiatry. (2020) 63:e32. 10.1192/j.eurpsy.2020.35 32234102 PMC7156565

[9] Torales J O'Higgins M Castaldelli-Maia JM Ventriglio A. The outbreak of COVID-19 coronavirus and its impact on global mental health. Int J Soc Psychiatry. (2020) 66:317–20. 10.1177/0020764020915212 32233719

[10] Lee J. Mental health effects of school closures during COVID-19. Lancet Child Adol Health. (2020) 2019:30109. 10.1016/S2352-4642(20)30109-7 32302537 PMC7156240

[11] Fegert JM Vitiello B Plener PL Clemens V. Challenges and burden of the coronavirus 2019 (COVID-19) pandemic for child and adolescent mental health: a narrative review to highlight clinical and research needs in the acute phase and the long return to normality. Child Adol Psychiatry Mental Health. (2020) 14:1–11. 10.1186/s13034-020-00329-3 PMC7216870 32419840

[12] Martin A Volkmar F. Lewis's Child and Adolescent Psychiatry: A Comprehensive Textbook. 4th ed. St. Philadelphia: Lippincot Williams and Wilkins (2007).

[13] Steinberg L. Adolescence. 11th ed. St. New York, NY: McGraw-Hill Education (2017).

[14] Oberle E Schonert-Reichl KA Thomson KC. Understanding the link between social and emotional well-being and peer relations in early adolescence: gender-specific predictors of peer acceptance. J Youth Adol. (2010) 39:1330–42. 10.1007/s10964-009-9486-9 20091211

[15] Grubic N Badovinac S Johri AM. Student mental health in the midst of the COVID-19 pandemic: a call for further research and immediate solutions. Int J Soc Psychiatry. (2020) 66:517–8. 10.1177/0020764020925108 32364039 PMC7405631

[16] Cao W Fang Z Hou G Han M Xu X Dong J. The psychological impact of the COVID-19 epidemic on college students in China. Psychiatry Res. (2020) 287:112934. 10.1016/j.psychres.2020.112934 32229390 PMC7102633

[17] Telkomsel. Trafik Jaringan Dan Layanan Komunikasi Berbasis Broadband Pelanggan Telkomsel Meningkat Hingga 16%. (2020). Available online at: https://www.telkomsel.com//about-us/news/trafik-jaringan-dan-layanan-komunikasi-berbasis-broadband-pelanggan-telkomsel (accessed Jun 20, 2020).

[18] The Jakarta Post. Telkomsel Reports 16 Percent Jump in Broadband Traffic as People Follow Physical Distancing Rules. (2020). Available online at: https://www.thejakartapost.com/news/2020/04/07/telkomsel-reports-16-percent-jump-in-broadband-traffic-as-people-follow-physical-distancing-rules.html (accessed July 1, 2020).

[19] Ni MY Yang L Leung CMC Li N Yao XI Wang Y. Mental health, risk factors, and social media use during the COVID-19 epidemic and cordon sanitaire among the community and health professionals in Wuhan, China: cross-sectional survey. JMIR Mental Health. (2020) 7:e19009. 10.2196/19009 32365044 PMC7219721

[20] Király O Potenza MN Stein DJ King DL Hodgins DC Saunders JB. Preventing problematic internet use during the COVID-19 pandemic: consensus guidance. Com Psychiatry. (2020) 100:152180. 10.1016/j.comppsych.2020.152180 32422427 PMC7215166

[21] Gao J Zheng P Jia Y Chen H Mao Y Chen S. Mental health problems and social media exposure during COVID-19 outbreak. PLoS ONE. (2020) 15:e0231924. 10.1371/journal.pone.0231924 32298385 PMC7162477

[22] Bahn GH. Coronavirus disease 2019, school closures, and children's mental health. J Korean Academy Child Adol Psychiatry. (2020) 31:74–9. 10.5765/jkacap.200010 32595345 PMC7289477

[23] World Health Organization. Adolescent Mental Health. (2020). Available online at: https://www.who.int/news-room/fact-sheets/detail/adolescent-mental-health (accessed January 20, 2021).

[24] Pardede N. Adolescence. In: Narendra MB Sularyo TS Soetjiningsih Suyitno H Ranuh IGNG, editors. Handb1ook of Child and Adolescent Development. 1st ed. St. Jakarta: Sagung Seto (2008). p. 139.

[25] Goodman A Goodman R. Strengths and difficulties questionnaire as a dimensional measure of child mental health. J Am Acad Child Adol Psychiatry. (2009) 48:400–3. 10.1097/CHI.0b013e3181985068 19242383

[26] Brann P Lethbridge MJ Mildred H. The young adult strengths and difficulties questionnaire (SDQ) in routine clinical practice. Psychiatry Res. (2018) 264:340–5. 10.1016/j.psychres.2018.03.001 29674224

[27] Oktaviana M Wimbarti S. Validasi klinik strength and difficulties questionnaire (SDQ) sebagai instrumen skrining gangguan tingkah laku. J Psikol. (2014) 41:101. 10.22146/jpsi.6961

[28] Goodman A Lamping DL Ploubidis GB. When to use broader internalising and externalising subscales instead of the hypothesised five subscales on the strengths and difficulties questionnaire (SDQ): data from british parents, teachers and children. J Abnor Child Psychol. (2010) 38:1179–91. 10.1007/s10802-010-9434-x 20623175

[29] Wiguna T Manengkei P Pamela C Rheza A Hapsari W. Masalah emosi dan perilaku pada anak dan remaja di poliklinik jiwa anak dan remaja RSUPN dr. Ciptomangunkusumo (RSCM). Jakarta Sari Pediatri. (2016) 12:270–4. 10.14238/sp12.4.2010.270-7

[30] Buysse DJ Reynolds CF Monk TH Berman SR Kupfer DJ. The pittsburgh sleep quality index: a new instrument for psychiatric practice and research. Psychiatry Res. (1989) 28:193–213. 10.1016/0165-1781(89)90047-4 2748771

[31] Mollayeva T Thurairajah P Burton K Mollayeva S Shapiro CM Colantonio A. The pittsburgh sleep quality index as a screening tool for sleep dysfunction in clinical and non-clinical samples: a systematic review and meta-analysis. Sleep Med Rev. (2016) 25:52–73. 10.1016/j.smrv.2015.01.009 26163057

[32] Alim I Winarsih S Elvira S. Uji Validitas Dan Reliabilitas Instrumen Pittsburgh Sleep Quality Index Versi Bahasa Indonesia. Jakarta: Universitas Indonesia (2015).

[33] Kircaburun K Griffiths MD Billieux J. Psychosocial factors mediating the relationship between childhood emotional trauma and internet gaming disorder: a pilot study. Eur J Psychotraumatol. (2019) 10:1565031. 10.1080/20008198.2018.1565031 30693081 PMC6338260

[34] Hayes AF Preacher KJ. Statistical mediation analysis with a multicategorical independent variable. Bri J Mathem Statis Psychol. (2014) 67:451–70. 10.1111/bmsp.12028 24188158

[35] Hu L Bentler PM. Cutoff criteria for fit indexes in covariance structure analysis: conventional criteria versus new alternatives. Str Equ Model. (1999) 6:1–55. 10.1080/10705519909540118

[36] Siste K Hanafi E Sen LT Christian H Adrian Siswidiani LP. The impact of physical distancing and associated factors towards internet addiction among adults in Indonesia during COVID-19 pandemic: a nationwide web-based study. Front Psychiatry. (2020) 11:580977. 10.3389/fpsyt.2020.580977 33101092 PMC7495250

[37] Lin M-P. Prevalence of internet addiction during the COVID-19 outbreak and its risk factors among junior high school students in Taiwan. IJERPH. (2020) 17:8547. 10.3390/ijerph17228547 33218018 PMC7698622

[38] Dong H Yang F Lu X Hao W. Internet addiction and related psychological factors among children and adolescents in China during the coronavirus disease 2019 (COVID-19). Epidemic Front Psychiatry. (2020) 11:751. 10.3389/fpsyt.2020.00751 32982806 PMC7492537

[39] Chia DXY Ng CWL Kandasami G Seow MYL Choo CC Chew PKH. Prevalence of internet addiction and gaming disorders in Southeast Asia: A meta-analysis. Int. J. Environ. Res. Public Health. (2020) 17:2582. 10.3390/ijerph17072582 32283803 PMC7177828

[40] Mihara S Osaki Y Nakayama H Sakuma H Ikeda M Itani O. Internet use and problematic internet use among adolescents in Japan: a nationwide representative survey. Addict Behav Rep. (2016) 4:58–64. 10.1016/j.abrep.2016.10.001 29511725 PMC5836521

[41] Park SK Kim JY Cho CB. Prevalence of internet addiction and correlations with family factors among South Korean adolescents. Adolescence. (2008) 43:895–909. 19149152

[42] Chang F-C Chiu C-H Lee C-M Chen P-H Miao N-F. Predictors of the initiation and persistence of internet addiction among adolescents in Taiwan. Addict Behav. (2014) 39:1434–40. 10.1016/j.addbeh.2014.05.010 24930050

[43] Wu X-S Zhang Z-H Zhao F Wang W-J Li Y-F Bi L. Prevalence of internet addiction and its association with social support and other related factors among adolescents in China. J Adol. (2016) 52:103–11. 10.1016/j.adolescence.2016.07.012 27544491

[44] Sigfusdottir ID Kristjansson AL Thorlindsson T Allegrante JP. Stress and adolescent well-being: the need for an interdisciplinary framework. Health Pro Int. (2017) 32:1081–90. 10.1093/heapro/daw038 27153917 PMC5914452

[45] Wu J Tong H Liu Z Tao J Chen L Chan CCH. Neurobiological Effects of Perceived Stress are Different Between Adolescents and Middle-Aged Adults. (2020). Available online at: http://link.springer.com/10.1007/s11682-020-00294-7 (accessed January 20, 2021). 10.1007/s11682-020-00294-7 PMC8032601 32737826

[46] Ferrara P Corsello G Ianniello F Sbordone A Ehrich J Giardino I. Internet addiction: starting the debate on health and well-being of children overexposed to digital media. J Pediatr. (2017) 191:280–1.e1. 10.1016/j.jpeds.2017.09.054 29637892

[47] Li W O'Brien JE Snyder SM Howard MO. Characteristics of internet addiction/pathological internet use in U.S. University Students: A Qualitative-Method Investigation. PLoS ONE. (2015) 10:e0117372. 10.1371/journal.pone.0117372 25647224 PMC4315426

[48] Müller J. Internet Usage in Singapore - Statistics & Facts. Statista. (2021). Available online at: https://www.statista.com/topics/5852/internet-usage-in-singapore/ (accessed January 30, 2021).

[49] Ni X Yan H Chen S Liu Z. Factors influencing internet addiction in a sample of freshmen university students in China. Cyberpsychol Behav. (2009) 12:327−330. 10.1089/cpb.2008.0321 19445631

[50] Ghamari F Mohammadbeigi A Mohammadsalehi N Hashiani AA. Internet addiction and modeling its risk factors in medical students, iran. Ind J Psychol Med. (2011) 33:158–62. 10.4103/0253-7176.92068 22345841 PMC3271491

[51] Tsitsika A Janikian M Schoenmakers TM Tzavela EC Olafsson K Wójcik S. Internet addictive behavior in adolescence: a cross-sectional study in seven European countries. Cyberpsychol Behav Soc Netw. (2014) 17:528–35. 10.1089/cyber.2013.0382 24853789

[52] Anderson J Rainie L. Millennials Will Make Online Sharing in Networks a Lifelong Habit. (2010). Available online at: http://pewresearch.org/millennials (accessed January 19, 2021).

[53] Effatpanah M Moharrami M Rajabi Damavandi G Aminikhah M Hosein Nezhad M Khatami F. Association of internet addiction with emotional and behavioral characteristics of adolescents. Iran J Psychiatry. (2020) 15:55–66. 10.18502/ijps.v15i1.2440 32377215 PMC7193233

[54] Cimino S Cerniglia L. A longitudinal study for the empirical validation of an etiopathogenetic model of internet addiction in adolescence based on early emotion regulation. Bio Med Res Int. (2018) 2018:4038541. 10.1155/2018/4038541 29707569 PMC5863349

[55] Wartberg L Kriston L Kramer M Schwedler A Lincoln TM Kammerl R. Internet gaming disorder in early adolescence: associations with parental and adolescent mental health. Eur Psychiatry. (2017) 43:14–8. 10.1016/j.eurpsy.2016.12.013 28365463

[56] Siste K. Development of Kuesioner Diagnostik Adiksi Internet for Adolescents: Brain Functional Connectivity Through fMRI BOLD, Study of Prevalence, Risk Factors, and Protective Factors. Jakarta: Universitas Indonesia (2019).

[57] Sugaya N Shirasaka T Takahashi K Kanda H. Bio-psychosocial factors of children and adolescents with internet gaming disorder: a systematic review. Bio Psycho Soc Med. (2019) 13:3. 10.1186/s13030-019-0144-5 30809270 PMC6374886

[58] Hu J Zhen S Yu C Zhang Q Zhang W. Sensation seeking and online gaming addiction in adolescents: a moderated mediation model of positive affective associations and impulsivity. Front Psychol. (2017) 8:699. 10.3389/fpsyg.2017.00699 28529494 PMC5418345

[59] Fernandes B Biswas UN Tan-mansukhani R Vallejo A Essau CA. The impact of COVID-19 lockdown on internet use and escapism in adolescents. Revistade Psicologí*a Cl*í*nica Niñosy Adolescentes*. (2020) 7:59–65. 10.21134/rpcna.2020.mon.2056

[60] Hertz MF Barrios LC. Adolescent mental health, COVID-19, and the value of school-community partnerships. Inj Prev. (2021) 27:85–6. 10.1136/injuryprev-2020-044050 33172840

[61] Ozbay F Johnson DC Dimoulas E Morgan CA Charney D Southwick S. Social support and resilience to stress: from neurobiology to clinical practice. Psychiatry. (2007) 4:35–40. 20806028 PMC2921311

[62] Wartberg L Brunner R Kriston L Durkee T Parzer P Fischer-Waldschmidt G. Psychopathological factors associated with problematic alcohol and problematic internet use in a sample of adolescents in Germany. Psychiatry Res. (2016) 240:272–7. 10.1016/j.psychres.2016.04.057 27138817

[63] Shek DTL Yu L. Adolescent internet addiction in Hong Kong: prevalence, change, and correlates. J Pediatr Adol Gynecol. (2016) 29(Suppl. 1):S22–30. 10.1016/j.jpag.2015.10.005 26461526

[64] Cao F Su L. Internet addiction among Chinese adolescents: prevalence and psychological features. Child Care Health Dev. (2007) 33:275–81. 10.1111/j.1365-2214.2006.00715.x 17439441

[65] Hou H Jia S Hu S Fan R Sun W Sun T. Reduced striatal dopamine transporters in people with internet addiction disorder. J Biomed Biotechnol. (2012) 2012:854524. 10.1155/2012/854524 22505818 PMC3312312

[66] Tian M Chen Q Zhang Y Du F Hou H Chao F. PET imaging reveals brain functional changes in internet gaming disorder. Eur J Nuclear Med Mol Imaging. (2014) 41:1388–97. 10.1007/s00259-014-2708-8 24737115

[67] Lobel A Engels RCME Stone LL Burk WJ Granic I. Video gaming and children's psychosocial well-being: a longitudinal study. J Youth Adol. (2017) 46:884–97. 10.1007/s10964-017-0646-z 28224404 PMC5346125

[68] Coyne SM Warburton WA Essig LW Stockdale LA. Violent video games, externalizing behavior, and prosocial behavior: a five-year longitudinal study during adolescence. Dev Psychol. (2018) 54:1868–80. 10.1037/dev0000574 30234338

[69] Jiang X Jiang W Cai J Su Q Zhou Z He L. Characterizing media content and effects of organ donation on a social media platform: content analysis. J Med Int Res. (2019) 21:e13058. 10.2196/13058 30860489 PMC6434401

[70] Myrick JG Willoughby JF. The role of media-induced nostalgia after a celebrity death in shaping audiences' social sharing and prosocial behavior. J Health Commun. (2019) 24:461–8. 10.1080/10810730.2019.1609140 31033409

[71] Franceschini C Musetti A Zenesini C Palagini L Scarpelli S Quattropani MC. Poor sleep quality and its consequences on mental health during the COVID-19 lockdown in Italy. Front Psychol. (2020) 11:3072. 10.3389/fpsyg.2020.574475 33304294 PMC7693628

[72] Lin P Lee Y Chen K Hsieh P Yang S Lin Y. The relationship between sleep quality and internet addiction among female college students. (2019) 13:1–9. 10.3389/fnins.2019.00599 PMC6582255 31249504

[73] Younes F Halawi G Jabbour H Osta N El Karam L Hajj A. Internet addiction and relationships with insomnia, anxiety, depression, stress and self-esteem in university students : a cross- sectional designed study. (2016) 11:e0161126. 10.1371/journal.pone.0161126 27618306 PMC5019372

[74] Smith WG. Does Gender Influence Online Survey Participation? A Record-Linkage Analysis of University Faculty Online Survey Response Behavior. (2008). Available online at: https://eric.ed.gov/?id=ED501717 (accessed March 13, 2021).

